# Evaluating the Effects of Prostate Radiotherapy Intensified with Pelvic Nodal Radiotherapy and Androgen Deprivation Therapy on Myelosuppression: Single-Institution Experience

**DOI:** 10.3390/curroncol31090402

**Published:** 2024-09-13

**Authors:** Yousef Katib, Steven Tisseverasinghe, Ian J. Gerard, Benjamin Royal-Preyra, Ahmad Chaddad, Tania Sasson, Boris Bahoric, Federico Roncarolo, Tamim Niazi

**Affiliations:** 1 College of Medicine, Taibah University, Madinah 42353, Saudi Arabia; 2 Department of Radiation Oncology, McGill University Health Centre, Montreal, QC H3A 3J1, Canada; steven.tisseverasinghe.med@ssss.gouv.qc.ca (S.T.); ian.gerard@mail.mcgill.ca (I.J.G.); benpreyra@gmail.com (B.R.-P.); 3 School of Artificial Intelligence, Guilin University of Electronic Technology, Guilin 541004, China; ahmad8chaddad@gmail.com; 4 Faculty of Medicine, McGill University, Montreal, QC H3A 0G4, Canada; 5 Department of Oncology, Jewish General Hospital, McGill University, Montreal, QC H3T 1E2, Canada; tania.sasson@mail.mcgill.ca (T.S.); bbahoric@jgh.mcgill.ca (B.B.); 6 School of Public Health, Université de Montréal, Montreal, QC H3N 1X9, Canada; federicoroncarolo@gmail.com

**Keywords:** prostate cancer, androgen deprivation therapy, myelosuppression, radiation therapy

## Abstract

Background: Prostate cancer (PCa) management commonly involves the utilization of prostate radiotherapy (PRT), pelvic nodal radiotherapy (PNRT), and androgen deprivation therapy (ADT). However, the potential association of these treatment modalities with bone marrow (BM) suppression remains inadequately reported in the existing literature. This study is designed to comprehensively evaluate the risk of myelosuppression associated with PRT, shedding light on an aspect that has been underrepresented in prior research. Materials and Methods: We conducted a retrospective analysis of 600 patients with prostate cancer (PCa) treated with prostate radiotherapy (PRT) at a single oncology center between 2007 and 2017. Patients were categorized into four cohorts: PRT alone (n = 149), PRT + ADT, (n = 91), PRT + PNRT (n = 39), and PRT + PNRT + ADT (n = 321). To assess the risk of myelosuppression, we scrutinized specific blood parameters, such as hemoglobin (HGB), white blood cells (WBCs), neutrophils (NEUT), lymphocytes (LYM), and platelets (PLT) at baseline, mid-treatment (mRT), immediately post-RT (pRT), 1 month post-RT (1M-pRT), and 1 year post-RT (1Y-pRT). The inter-cohort statistical significance was evaluated with further stratification based on the utilized RT technique {3D conformal radiotherapy (3D-CRT), and intensity-modulated radiation therapy (IMRT)}. Results: Significant statistical differences at baseline were observed in HGB and LYM values among all cohorts (*p* < 0.05). Patients in the PRT + PNRT + ADT cohort had significantly lower HGB at baseline and 1M-pRT. In patients undergoing ADT, BMS had a significant impact at 1M-pRT {odds ratio (OR) 9.1; 95% Confidence Interval (CI) 4.8–17.1} and at 1Y-pRT (OR 2.84; CI 1.14–7.08). The use of 3D-CRT was linked to reduced HGB levels in the PRT + PNRT + ADT group at 1 month pRT (*p* = 0.015). Similarly, PNRT significantly impacted BMS at 1M-pRT (OR 6.7; CI 2.6–17.2). PNRT increased the odds of decreased WBC counts at 1Y-pRT (OR 6.83; CI: 1.02–45.82). Treatment with any RT techniques (3D-CRT or IMRT), particularly in the PRT + PNRT and PRT + PNRT + ADT groups, significantly increased the odds of low LYM counts at all time points except immediately pRT (*p* < 0.05). Furthermore, NEUT counts were considerably lower at 1M-pRT (*p* < 0.05) in the PRT + PNRT + ADT group. PLT counts were significantly decreased by PRT + PNRT + ADT at mRT (OR 2.57; 95% CI: 1.42–4.66) but were not significantly impacted by the RT technique. Conclusions: Treatment with PRT, ADT, PNRT, and 3D-CRT is associated with BMS. Despite this statistically significant risk, no patient required additional interventions to manage the outcome. While its clinical impact appears limited, its importance cannot be underestimated in the context of increased integration of novel systemic agents with myelosuppressive properties. Longer follow-up should be considered in future studies.

## 1. Introduction

Prostate cancer (PCa) stands as the second most prevalent cancer in men and ranks as the fifth leading cause of cancer-related fatalities worldwide. In 2020, there were approximately 1.4 million new cases reported globally [1]. In the United States during the same year, approximately 192,000 men were diagnosed with PCa, and 33,000 succumbed to the disease [2]. For early-stage high-risk cancers characterized by a Gleason score ≥ 8, stage ≥ T3a, PSA ≥ 20 or ISUP grade group ≥ 4, definitive radiotherapy (RT) remains an excellent treatment option. Although nearly 80% of patients can experience remission, recurrence may occur in up to 55% of cases [3]. Indeed, nearly 12% of PCa patients develop lymph node or distant metastasis, which can drastically alter their survival and often render further curative treatment modalities ineffective [4]. About one-third of patients opt for external beam radiation therapy (EBRT) or brachytherapy (BT) as their preferred treatment choice. However, despite improved conformality, dose escalation and a better understanding of ADT synergy, biochemical failure may still occur [5]. In specific patient subsets, the addition of ADT to RT has shown improvement in progression-free survival (PFS) and overall survival (OS), notably in the high-risk population [6]. While ADT yields favorable prognostic effects, it also negatively impacts BM activity, increasing the risk of hematological toxicity, such as myelosuppression [7,8,9,10,11]. Unfortunately, relapse rates while on ADT are quite high, with a median time to failure of approximately 11 months [12].

Hematopoietic stem cells, sensitive to radiation, continuously replace mature peripheral blood cells [8]. Radiation doses of 2–4 Gy for 1–3 days can reduce BM cellularity, while a total dose of 30–40 Gy can achieve complete ablation of the BM [13,14,15]. The extent of myelosuppression is influenced by the total volume of irradiated BM [16]. Approximately 55% of adult hematopoietic stem cell production occurs within the BM, distributed among various bones, with up to 25% in pelvic bones alone [8,17]. During RT for high-risk PCa, PRT + PNRT can expose the sacrum, pelvic bones, and proximal femurs to radiation, leading to myelosuppression [18,19,20,21].

The RT technique significantly impacts the total dose received by normal pelvic tissue and the extent of BM irradiation, affecting myelosuppression [7,17]. Generally, IMRT (intensity-modulated radiation therapy) further reduces the dose to critical OARS through dose painting [22,23]. When compared to 3D-CRT, IMRT can achieve superior conformal target coverage, albeit at the expense of a higher integral dose borne by surrounding normal tissues. Hence, IMRT diminishes elevated doses to the adjacent pelvic normal tissues, showcasing fewer genitourinary or gastrointestinal-related adverse effects. IMRT creates a more conformal radiation dose distribution compared to 3D-CRT. Hence, a lower BM volume often receives a radiation dose that can cause myelosuppression with IMRT treatment compared to 3D-CRT [24,25,26]. Improvements in BM-sparing IMRT techniques are an ongoing and active area of research [27,28,29]. Numerous clinical trials have undertaken a comparative analysis of 3D-CRT and IMRT in the management of PCa [7,23,24,30]. However, uncertainty exists regarding the comparative difference of 3D-CRT versus IMRT on hematologic toxicity [17,23,24]. As of now, no published research has delved into the distinctions in hematologic toxicity between PRT alone and PNRT, with or without ADT. Consequently, through this study, we aim to meticulously evaluate the effects of the RT technique, RT volume, and ADT on hematologic toxicity in men undergoing treatment for PCa.

## 2. Materials and Methods

We conducted a large single-institution retrospective review of a cohort of 600 patients with localized prostate cancer (PCa) undergoing PRT ± PNRT ± ADT from 2007 to 2017. Radiation treatment plans utilized either a 3D-CRT or IMRT technique. PNRT encompassed coverage of the bilateral distal common iliac, external iliac, internal iliac, presacral, and obturator nodal regions. The treatment field was precisely delineated from the L5-S1 vertebral junction, superiorly to the caudal edge of the pubic symphysis, inferiorly. ADT comprised the administration of a luteinizing hormone-releasing hormone (LHRH) agonist with an oral anti-androgen (bicalutamide) for the first month. The duration of ADT varied between short-term (6 months) ADT for unfavorable intermediate-risk and long-term (24 months) for high-risk patients. The radiation dose ranged from moderate hypofractionation (60–66 Gray/20–23 fractions) or conventional fractionation (76–78 Gray in 38–39 fractions). The primary endpoint of this study was to evaluate the effect of treatment on BM activity in PCa patients, and to comprehensively assess the risk of myelosuppression through specific hematologic parameters. Initially, all patients were stratified per their treatment regimen into four cohorts: PRT alone (Cohort 1, n = 149), PRT and ADT (Cohort 2, n = 91), PRT and PNRT (Cohort 3, n = 39), and PRT, PNRT, and ADT (Cohort 4, n = 321). Subsequently, the hematologic parameters HGB, WBC, PLT, LYMPH, and NEUT counts were scrutinized at five time points: (1) at baseline (immediately before RT), (2) midway through RT (mRT), (3) immediately post-RT (pRT), (4) one month post-RT (1M-pRT), and one year post-RT (1Y-pRT). The numerical values of each parameter were converted into binary values (normal or abnormal). Abnormal values were defined as those falling below the threshold of normal for a healthy male adult: HGB < 140 g/L, PLT < 150 × 10^9^/L, LYMPH < 1.2 × 10^9^/L, WBC < 4 × 10^9^/L, and NEUT < 1.8 × 10^9^/L. To assess changes in hematologic parameters over time, patients were categorized into one of four states during follow-up at the four predefined time points: (a) normal values remaining normal, (b) abnormal values becoming normal, (c) normal values becoming abnormal, and (d) abnormal values remaining abnormal.

The comparisons between cohorts for each hematologic parameter were conducted at the five predefined time points using Pearson’s Chi-Squared tests. Furthermore, each hematologic parameter was evaluated using multiple regression models at each predefined time point. The first regression model examined the probability of hematologic parameters that were normal before treatment becoming abnormal at the predefined time point, with PRT as the comparator. The second regression model assessed the likelihood of abnormal hematologic parameters in each cohort becoming normal. The covariate in both regression models was the radiotherapy (RT) technique used for planning, specifically 3D-CRT vs. IMRT. All data analyses were executed with the IBM SPSS 21.0 program (SPSS, Inc., Chicago, IL, USA).

## 3. Results

The mean patient age was 71.5 years (range: 52–86). Diabetes (n = 132, 22.3%), hypertension (n = 303, 51.2%), and hypercholesterolemia (n = 221, 37.3%) were the most frequent chronic illnesses identified in this cohort. Patients were stratified by NCCN risk groups into low-risk (n = 15, 2.5%), intermediate-risk (n = 220, 36.6%), and high-risk (n = 365, 60.8%) categories. Only two patients were diagnosed with stage IV PCa (node-positive). Treatment modalities included 3DCRT in 28.5% (n = 171) and IMRT in 71.5% (n = 429) of cases. All patients with high-risk disease received pelvic RT except five patients due to technical challenges. Patients with low and int risk received PRT. Pelvic RT was given to 360 patients (60%), while PRT was used to treat 240 patients (40%). In addition, ADT was utilized in 412 patients (68.6%), of which 91 (24.8%) patients had intermediate-risk and 321 (53.3%) had high-risk PC. Baseline mean blood counts revealed an HGB value of 138.5, a NEUT count of 4.2, a LYMPH count of 1.8, and a PLT count of 207. Comprehensive demographic information is further detailed in Table 1. 

**Figure 1:**
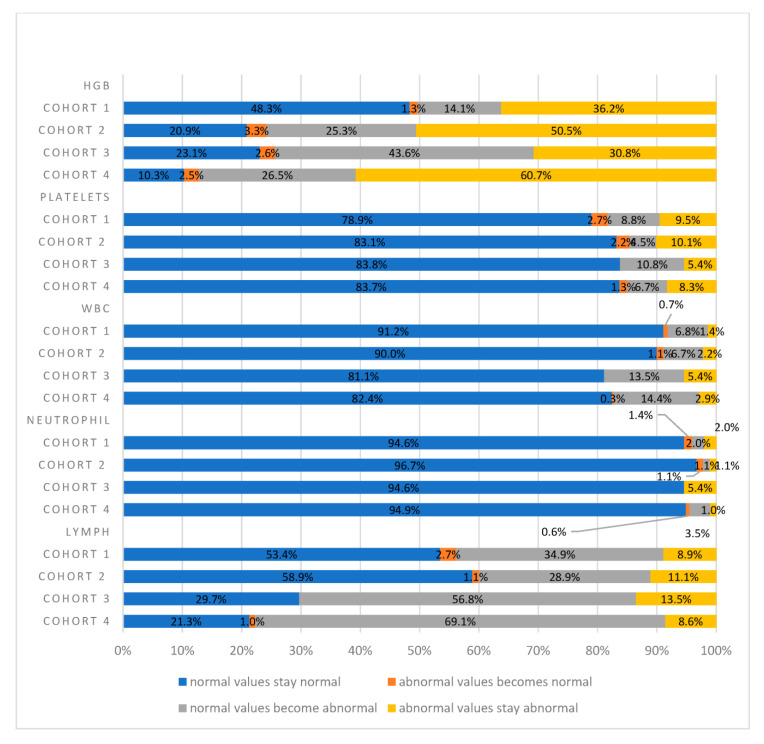
The effect of the different therapies on hematological parameters at 1m-pRT. Across the four cohorts, statistically significant differences (*p* < 0.05) were found for HGB and LYMPH values. Cohort 1: PRT alone; Cohort 2: PRT and ADT; Cohort 3: PRT and PNRT; Cohort 4: PRT, PNRT, and ADT.

Significant differences in hemoglobin (HGB) values were identified among all four cohorts at baseline (*p* < 0.05), as highlighted in Table 2. However, no other statistically significant differences were found at baseline for any other hematologic parameters across all cohorts. A decrease in HGB was consistently observed at various time points, reaching the mRT-pRT stages, with odds ratios (ORs) of 6.71 (CI 2.84–15.83), 5.11 (CI 1.94–13.44), and 9.52 (CI 4.7–19.04) for Cohorts 2, 3, and 4, respectively (Figure 1). Cohort 4 exhibited an increased likelihood of suffering from decreased HGB, with a 26.5% drop in values on average, and persisting in 60% of the cohort at every time point throughout the treatment course. In Cohort 3 (PRT + PNRT), there was a 43.6% probability of patients with initially normal HGB values transitioning to abnormally lower levels during the course of therapy, with 30.8% maintaining such levels thereafter. Notably, the likelihood of such transitions from normal to abnormal HGB values in Cohort 3 was lower compared to Cohorts 4 and 1, as illustrated in Figure 1.

These parameters were graded as per the Common Terminology Criteria for Adverse Effects (CTCAE) v4.0 (US Department of Health and Human Services, 2009). Significant differences among the four cohorts were found related to baseline hemoglobin levels (*p* < 0.05).

Furthermore, HGB values decreased in all cohorts, with odds ratios (ORs) of 8.80 (CI 3.36–23.03), 5.15 (CI 1.8–13.67), and 12.20 (CI 5.95–25.01) for Cohorts 2, 3, and 4, respectively. HGB values for Cohort 4 decreased the most at the pRT time point (*p* < 0.02). The ORs for decreased HGB at 1M-pRT were 4.08 (CI 1.87–8.91), 6.7 (CI 2.6–17.2), and 9.1 (CI 4.8–17.1) for Cohorts 2, 3, and 4, respectively. At 1Y-pRT, The HGB for all cohorts approached baseline without any significant differences, except for Cohort 4, where a residual OR of 2.84 (CI 1.14–7.08) persisted.

Moreover, it was evident that decreased HGB values were more pronounced with IMRT compared to 3D-CRT, accounting for 14% across all four cohorts (Table 3). Cohort 3 also displayed a 43.6% likelihood of normal HGB values shifting to abnormal levels, which was then sustained at abnormal levels in 30.8% during therapy. Although the probability of HGB values transitioning from normal to abnormal was lower compared to Cohort 4, it was still considerable. 

In Cohort 4, a significant proportion of patients experienced a transition from normal HGB values to abnormal values post-radiotherapy (*p* < 0.02). The odds ratios for this transition at 1M-pRT were 4.08 (CI 1.87–8.91) for Cohort 2, 6.7 (CI 2.6–17.2) for Cohort 3, and 9.1 (CI 4.8–17.1) for Cohort 4. At 1Y-pRT, the odds ratio for Cohort 4 was 2.84 (CI 1.14–7.08), indicating a notable increase, although this change did not reach statistical significance for Cohorts 2 and 3.

**Figure 2:**
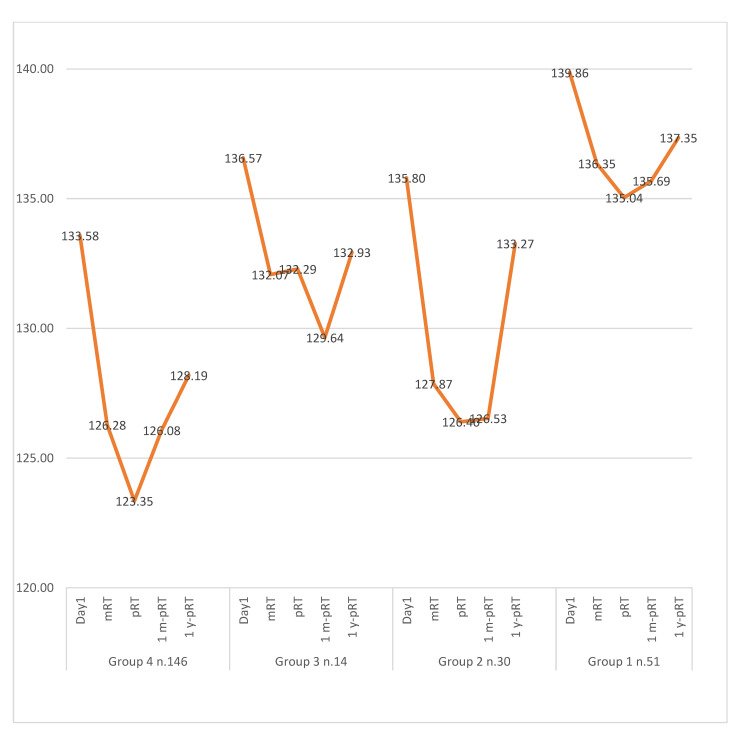
HGB values change over time. Here, it is shown how HGB values hit a peak at mRT and end of RT and then gradually recovered to baseline. Patients with incomplete data sets were excluded. Day1 = baseline; mRT = midway through RT; pRT = immediately pRT; 1mo-pRT = one month post-RT; 1y-pRT = one year post-RT.

HGB decreased in all cohorts with RT. However, the decrease was generally more pronounced with 3D-CRT than IMRT (Table 3). However, low HGB in Cohort 4 (63.7%) occurred more frequently with IMRT than with 3D-CRT (Table 3). A comprehensive overview of mean HGB levels across cohorts over time is presented in Table 2 and Figure 2. The temporal evolution of other hematologic parameters is delineated in Figure 2.

Our analysis also delved into alterations in WBC and NEUT values. Patients within Cohort 4 exhibited a heightened probability (14.4%) of transitioning from normal WBC values to abnormal values at every time point (Figure 1). Notably, this trend was not evident in NEUT values. The only statistically significant instance of WBC values transitioning from normal to abnormal levels was seen at the mRT time point. For Cohorts 3 and 4, the odds ratios (ORs) stood at 4.02 (95% CI: 1.20–13.41) and 3.91 (95% CI: 1.62–9.43), respectively, with a *p*-value < 0.05 (refer to Table 4 and Table 5). This significant finding persisted at both the pRT and 1m-pRT time points in Cohort 4, revealing ORs of 2.39 (95% CI: 1.16–4.93) and 2.42 (95% CI: 1.18–99), respectively (see Table 4 and Table 5). Furthermore, a statistically significant change was noted at the 1Y-pRT time point in Cohort 3, with an OR of 6.83 (95% CI: 1.02–45.82). Conversely, no statistically significant differences were discerned in any NEUT comparisons among all cohorts at the predefined time points.

Significant shifts from normal to abnormal LYMPH values were observed in Cohort 4 at all predefined time points. The odds ratios (ORs) for these transitions were notably high: 8.24 (95% CI: 4.91–13.82), 6.42 (95% CI: 3.73–11.05), 5.12 (95% CI: 3.25–8.06), and 4.42 (95% CI: 1.79–7.04) at the mRT, pRT, 1m-pRT and 1y-pRT time points, respectively (Table 5). Notably, at baseline, there was a statistically significant difference in LYMPH values among all four cohorts (*p* < 0.05) (see Table 2). Patients in Cohort 4 and Cohort 3 demonstrated the highest likelihoods of transitioning from normal LYMPH values to abnormal values at every time point, with percentages of 69.1% and 56.8%, respectively, depicted in Figure 1. In contrast, the probability of LYMPH values transitioning from normal to abnormal was lower in Cohort 1 and Cohort 2. Abnormal LYMPH values displayed sustained abnormality across all four cohorts, as illustrated in Figure 1. For the same hematologic parameter, Cohort 3 reached statistical significance at all predefined time points except at 1y-pRT, with ORs of 8.37 (95% CI: 2.76–25.46), 3.52 (95% CI: 1.24–9.97), and 3.03 (95% CI: 1.34–6.8) for the mRT, pRT, and 1m-pRT time points, respectively (see Table 5). In contrast, Cohort 2 did not attain statistical significance at any point in time. In patients receiving Post-Neoadjuvant Radiotherapy (PNRT) in Cohorts 3 and 4, more than 50% of LYMPH values shifted from normal to abnormal at the 1m-pRT time point. Additionally, LYMPH values were significantly lower in the 3D-CRT group, at baseline, mRT, 1m-pRT, and 1y-pRT (*p* < 0.05).

The only significant decrease in PLT values was observed exclusively at the mRT time point for Cohort 4, accounting for 6.7% of cases. This change was statistically significant, reflected by an odds ratio (OR) of 2.57 (95% CI: 1.42–4.66) as outlined in Table 5. Conversely, no other comparisons for PLT values reached statistical significance. In Cohorts 1, 2, and 3, the majority of PLT values were within the normal range, and this normalcy persisted after RT, as depicted in Figure 1.

## 4. Discussion

Hematologic toxicities (HTs), such as leukopenia, thrombocytopenia, and anemia, are common and potentially life-threatening side effects of oncologic treatments for pelvic malignancies. Specifically, these toxicities have been frequently associated with PCa treatment involving radiation therapy (RT) that includes bone marrow irradiation. Despite this association, the clinical impact of RT-related HTs in PCa patients remains unclear. Our study aimed to address this gap by assessing the impact of pelvic radiation therapy, with or without ADT, on the risk of myelosuppression in PCa patients treated with various radiotherapy modalities.

In our study, 600 PCa patients were categorized based on their treatment modality into four groups receiving RT at predefined time points. Among them, 365 high-risk PCa patients underwent PRT, pelvic nodal radiation therapy (PNRT), and long-term ADT for up to 2 years, while 205 intermediate-risk PCa patients received PRT with or without short-term ADT for up to 6 months. Additional groups included favorable intermediate-risk PCa (n = 15) and low-risk PCa (n = 15) patients treated with isolated PRT (refer to Table 1). Notably, a significant integral dose was delivered to pelvic bones, sacrum, and proximal femurs for most patients, likely contributing to the observed results. However, no patient in any group required medical intervention for myelosuppression at predefined time points. Our findings indicated a negative association between PNRT, ADT, and myelosuppression. The impact of RT on bone marrow (BM) activity in Cohorts 1 and 2 was minor compared to Cohorts 3 and 4. Across all cohorts, more than 80% of patients exhibited normal platelet (PLT), white blood cell (WBC), and neutrophil (NEUT) counts at 1M-pRT. However, those undergoing radiotherapy, including PNRT, demonstrated the most significant impact on BM activity. When assessing the risk of BM suppression in hematological parameters, HGB count was the most affected value, followed by WBC and LYMPH counts (refer to Figure 1). Our findings align with existing literature, indicating that the combination of RT and ADT in PCa treatment leads to statistically significant reductions in HGB and LYMPH values. Among the cohorts, those receiving PNRT experienced the most pronounced impact, likely due to the higher integral dose received in the BM [19,31,32,33]. In a study by Ikushima et al., 158 women with pelvic cancers underwent a course of radiotherapy [32]. The authors found an increase in pelvic insufficiency fractures in regions exposed to radiation, which was likely attributable to the impact of radiation on bone marrow (BM) activity. Similarly, Okonogi et al. observed decreases in bone marrow density in previously irradiated regions one year post-treatment [31]. While these investigations did not focus on patients with PCa, they highlight the impact of radiotherapy on BM density and composition. Prospective research exploring the reduction in one-year dose to the bone marrow through advanced radiation planning techniques, such as proton therapy, holds promise in identifying strategies to mitigate the impact of RT on myelosuppression. However, it is crucial to note that the clinical significance of myelosuppression in this context has not been thoroughly characterized. Future studies should delve deeper into understanding its implications in terms of health-related and overall quality of life. 

Our study uncovered a correlation between the type of radiation therapy (RT) technique used (3D-CRT versus IMRT) and the risk of myelosuppression. Notably, patients in Cohort 4 treated with IMRT exhibited greater odds of HGB deficiency, with abnormal HGB counts observed in 64% of exposed patients, more so than those treated with 3D-CRT (refer to Table 3). This might perhaps explain that a more widespread low integral dose may have a greater impact on bone marrow already partially compromised from ADT. Both Erpolat et al. and Avinash et al. demonstrated that IMRT reduces irradiated bone marrow (BM) volumes more effectively than 3DCRT. However, no significant difference between the two techniques was observed in terms of acute and chronic hematologic toxicities [7]. It is worth noting that IMRT alone may not be the sole cause of acute hematotoxicities in these patients; platinum-based chemotherapy might contribute as a coexisting factor [7,34]. Despite not requiring medical intervention for myelosuppression following RT, further investigation is crucial to assess the clinical significance of the hematologic findings identified in our study, especially in terms of their impact on quality of life and disease outcomes. Acknowledged limitations in our study include its retrospective nature, differences in reporting practices among pathologists, and variations in practice regarding neoadjuvant systemic therapies. Additionally, the exclusion of patients undergoing brachytherapy limits the generalizability of our findings to this population. In Cohorts 2 and 4, 50% of patients had abnormal HGB at baseline, possibly confounding the analysis. In Cohort 2, the likelihood of normal HGB values shifting to abnormal closely resembled that of Cohort 4, which could be related to the effect of ADT.

## 5. Conclusions

Our study has demonstrated that treatment with PRT, PNRT, ADT, and IMRT can substantially impact the risk of myelosuppression. It is noteworthy that myelosuppression induced by RT and ADT is generally inconsequential and temporary, as the majority of patients return to baseline within four weeks post-PRT. Importantly, despite the acknowledged risks, none of the patients required additional interventions to mitigate the outcomes.

To bolster the thoroughness of forthcoming studies, it is advisable to extend the duration of follow-up periods and integrate patient-reported outcomes. This methodological enhancement will yield valuable insights into the clinical significance of observed changes and their potential long-term effects. The exploration of novel technologies shows potential in enhancing the therapeutic ratio by mitigating treatment-associated toxicity. Sustained research efforts in these domains are vital for the continued refinement of treatment strategies and improvements in patient safety.

## Figures and Tables

**Table 1:** Clinical and biological demographic data of the 600 PCa patients involved in this study.

	Frequency n/(%)
Age	
Mean	71.5
Range	52–86
Associated Chronic Diseases	
DM	
Yes	132 (22.3%)
No	460 (77.7%)
HTN	
Yes	303 (51.2%
No	289 (48.8%)
Cholesterol	
Yes	221 (37.3%)
No	371 (62.7%)
AJCC Tumor Stage (n = 600)	
- T1	194 (32.3%)
- T2	212 (35.3%)
- T3	167 (27.8%)
- T4	004 (0.7%)
- Unknown	021 (3.5%)
- N0	598 (99.7%)
- N1	2 (0.3%)
Global stage	
- IV	2 (0.3%)
Gleason score	
- ≤6	041 (6.9%)
- 7	281 (47.1%)
- ≥8	275 (46.1%)
NCCN risk group	
- Low	015 (2.5%)
- Intermediate	220 (36.6%)
. Favorable	129 (21.5%)
. Unfavorable	91 (15.1%)
- High	365 (60.8%)
Treatment techniques	
- 3D-CRT (Patient #)	171 (28.5%)
- IMRT	429 (71.5%)
Treatment site	
- Pelvic RT	360 (60%)
- Prostate RT	240 (40%)
LHRH	
- Yes	412 (68.7%)
- No	188 (31.3%)
Type of treatment	
Cohort 1: Prostate radiation only	149 (24.8%)
Cohort 2: Prostate radiation and ADT	091 (24.8%)
Cohort 3: Prostate and pelvic radiation	039 (6.5%)
Cohort 4: prostate, pelvic radiation, and ADT	321 (53.5%)
Hematological Counts (median/range)	
Hemoglobin	138.5 (82–176)
Neutrophils	4.2 (0–16.9)
Lymphocytes	1.8 (0–30.4)
Platelets	207 (0–612)

**Table 2:** Baseline HGB values stratified by all four cohorts *.

	HGB (<140 g/L) *		HGB (≥140 g/L)		PLT (<150 × 10^9^/L)		PLT (≥150 × 10^9^/L)		WBC (<4 × 10^9^/L)		WBC (≥4 × 10^9^/L)		NEUT (<1.8 × 10^9^/L)		NEUT (≥1.8 × 10^9^/L)		LYMPH (<1.2 × 10^9^/L)		LYMPH (≥1.2 × 10^9^/L)	
	N.	%	N.	%	N.	%	N.	%	N.	%	N.	%	N.	%	N.	%	N.	%	N.	%
**Cohort 1**	56	37.6	93	62.4	18	12.1	131	87.9	4	2.7	145	97.3	5	3.4	144	96.6	17	11.4	132	88.6
**Cohort 2**	49	53.8	42	46.2	11	12.1	80	87.9	3	3.3	88	96.7	2	2.2	89	97.8	11	12.1	80	87.9
**Cohort 3**	13	33.3	26	66.7	3	7.7	36	92.3	2	5.1	37	94.9	2	5.1	37	94.9	5	12.8	34	87.2
Cohort 4 *	203	63.2	118	36.8	31	9.7	290	90.3	10	3.1	311	96.9	5	1.6	316	98.4	31	9.7	290	90.3
**Total**	321	53.5	279	46.5	63	10.5	537	89.5	19	3.2	581	96.8	14	2.3	586	97.7	64	10.7	536	89.3

* A statistically significant difference in HGb *p*-value compared to the baseline was observed in cohort 4.

**Table 3:** HGB changes over time according to the RT technique (3D-CRT vs. IMRT) for Cohort 4 at the mRT time point.

		Normal–Abnormal		Abnormal–Normal		Normal–Normal		Abnormal–Abnormal	
		n.	%	n.	%	n.	%	n.	%
Cohort 1	3dCRT	29	54.7	0	0	8	15.1	16	30.2
	IMRT	43	44.8	2	2.1	13	13.5	38	39.6
Cohort 2	3dCRT	7	17.5	2	5.0	8	20.0	23	57.5
	IMRT	12	23.5	1	2.0	15	29.4	23	45.1
Cohort 3	3dCRT	3	37.5	0	0	1	12.5	4	50.0
	IMRT	6	19.4	1	3.2	16	51.6	8	25.8
Cohort 4 *	3dCRT	5	7.1	1	1.4	29	41.4	35	50
	IMRT	28	11.2	7	2.8	56	22.3	160	63.7

* *p*-value for normal–normal vs. normal–abnormal is =0.04. *p*-value for abnormal values that continued to be abnormal vs. abnormal values that shifted to normal is =0.69.

**Table 4:** Hematologic parameter changes over time: values remaining abnormal 1m-pRT.

	HGB (<140 g/L)		PLT (<150 × 10^9^/L)		LYMPH (<1.2 × 10^9^/L)		WBC (<4 × 10^9^/L)		NEUT (<1.8 × 10^9^/L)	
	N.	%	N.	%	N.	%	N.	%	N.	%
Cohort 1	54	36.2	14	9.4	13	8.7	2	1.3	3	2
Cohort 2	46	50.2	9	9.9	10	11	2	2.2	1	1.1
Cohort 3	12	30.8	2	5.1	5	12.8	2	5.1	2	5.1
Cohort 4	195	60.7	26	8.1	27	8.4	9	2.8	3	0.9

**Table 5:** Summary of ORs for statistically significant relationships in primary regression analysis for a negative change (normal to abnormal) in each of the hematologic parameters, except NEUT as no statistically significant comparison was observed. mRT = midway through RT; pRT = immediately post-RT; 1m-pRT = one month post-RT; 1y-pRT = one year post-RT.

Cohort	OR (95% Confidence Interval)															
	HGB				WBT				PLT				LYMPH			
	**mRT**	**pRT**	**1m-pRT**	**1y-pRT**	**mRT**	**pRT**	**1m-pRT**	**1y-pRT**	**mRT**	**pRT**	**1m-pRT**	**1y-pRT**	**mRT**	**pRT**	**1m-pRT**	**1y-pRT**
Cohort 2	**6.71 (2.84–15.83)**	**8.80 (3.36–23.03)**	**4.08 (1.87–8.91)**	N/A		N/A	N/A	N/A	N/A	N/A	N/A	N/A	N/A	N/A	N/A	N/A
Cohort 3	**5.11 (1.94–13.44)**	**5.15 (1.8–13.67)**	**6.7 (2.6–17.2)**	N/A	**4.02 (1.20–13.41)**	N/A	N/A	**6.83 (1.02–45.82),**	N/A	N/A	N/A	N/A	**8.37 (2.76–25.46)**	**3.52 (1.24–9.97)**	**3.03 (1.34–6.8)**	N/A
Cohort 4	**9.52 (4.7–19.04)**	**12.20 (5.95–25.01)**	**9.1 (4.8–17.1)**	**2.84 (1.14–7.08)**	**3.91 (1.62–9.43)**	**2.39 (1.16–4.93).**	**2.42 (1.18–99)**	N/A	**2.57 (1.42–4.66).**	N/A	N/A	N/A	**8.24 (4.91–13.82**	**6.42 (3.73–11.05)**	**5.12 (3.25–8.06)**	**3.54 (1.79–7.04)**

We compare treatment in Cohort 1 to other cohort treatments.

## Data Availability

The data presented in this study are available on request from the corresponding authors.

## Abbreviations

PCa	Prostate cancer
PRT	Prostate radiotherapy
PNRT	Pelvic nodal radiotherapy
RT	Radiotherapy
ADT	Androgen deprivation therapy
LHRH	Luteinizing hormone-releasing hormone
3D-CRT	3D conformal radiotherapy
IMRT	Intensity-modulated radiotherapy
HGB	Hemoglobin
WBC	White blood cell
PLT	Platelets
NEUT	Neutrophils
LYMPH	Lymphocytes
BM	Bone marrow
CTCAE	Common Terminology Criteria for Adverse Effects
